# Large-Scale Crowd Analysis through the Use of Passive Radio Sensing Networks

**DOI:** 10.3390/s20092624

**Published:** 2020-05-04

**Authors:** Stijn Denis, Ben Bellekens, Abdil Kaya, Rafael Berkvens, Maarten Weyn

**Affiliations:** IDLab–Faculty of Applied Engineering, University of Antwerp–imec, Sint-Pietersvliet 7, 2000 Antwerp, Belgium; ben.bellekens@uantwerpen.be (B.B.); abdil.kaya@uantwerpen.be (A.K.); rafael.berkvens@uantwerpen.be (R.B.); maarten.weyn@uantwerpen.be (M.W.)

**Keywords:** device-free, radio frequency, RF, wireless sensor networks, WSN, passive sensing, sensorless sensing, crowd estimation, crowd counting, crowd analytics, footfall analytics

## Abstract

The creation of an automatic crowd estimation system capable of providing reliable, real-time estimates of human crowd sizes would be an invaluable tool for organizers of large-scale events, particularly so in the context of safety management. We describe a set of experiments in which we installed a passive Radio Frequency (RF) sensor network in different environments containing thousands of human individuals and discuss the accuracy with which the resulting measurements can be used to estimate the sizes of these crowds. Depending on the selected training approach, a median crowd estimation error of 184 people could be obtained for a large scale environment which contained 3227 people at its peak. Additionally, we look into the potential benefits of dividing one of our experimental environments into multiple subregions and open up a potentially interesting new topic of research regarding the estimation of crowd flows. Finally, we investigate the combination of our measurements with another sources of crowd-related data: sales data from drink stands within the environment. In doing so, we aim to integrate the concept of an automatic RF-based crowd estimation system into the broader domain of crowd analysis.

## 1. Introduction

Crowd management is an integral aspect of the organization of any type of large-scale event. Special attention needs to be paid to the layout of the event environment(s) in order to ensure the safety and comfort of the attendees and to decrease the risk of crowd disasters occurring. The number and locations of available entrances and exits, the placement of barriers to steer the crowd along desired routes and the use of clear signage to inform visitors where event activities are located, are but a few examples of elements which need to be taken into account [1].

While it is still far from common practice, there has been an increased tendency to make use of mathematical models in creating preparatory crowd management plans [2,3]. These models can be classified as being part of crowd science, an active field of research combining insights from mathematics, physics, psychology and sociology [3,4,5]. Furthermore, practical modelling tools have been developed which can aid experienced crowd managers within control centers in performing real-time risk assessments [6]. Crowd management is not limited to preparatory planning and event organizers must also be capable of detecting unexpected crowd-related occurrences and react in a timely and appropriate manner, often in cooperation with emergency services. In this context, real-time information regarding the density of crowds (crowd density estimation) and the number of people of which they are comprised (crowd counting) can be highly valuable. Multiple low- and high-tech solutions do exist to obtain this information, but they each have several major shortcomings.

One of the most common and simple methods consists of security personnel on the ground regularly providing crowd estimates. For comparatively simple environments with a limited number of manned entrances and exits, manual counting—potentially aided through the use of a tally counter—can prove to be sufficient. For more complex environments with less clearly delineated entrances and exits (e.g., a stage environment at a music festival), it can be very difficult to manually perform accurate crowd sizes estimates in large-scale environments containing thousands of human individuals and the numbers provided by different security agents are likely to diverge considerably. Camera systems are commonly used to provide a centralized overview of multiple environments in the control center. The resulting live images can then be analyzed by crowd experts, thereby lowering the amount of security personnel that needs to be deployed. With the strident advances made within the field of computer vision over the past few decades, these images can even be analyzed algorithmically [7,8], which (largely) removes the subjective human element. However, these systems tend to require vast amounts of available computing power [9]. Furthermore, the accuracy of these approaches can be influenced heavily by environmental aspects such as smoke, rapidly changing lighting conditions (a common occurrence at music festivals) and occlusion in general. Additionally, a major issue which needs to be kept in mind at all times when utilizing camera-based approaches, is privacy. In recent years, privacy in general is a topic which has received considerable interest, not just from a legal point of view, but also within the public consciousness.

An increasingly popular non-vision based approach is to make use of the Radio Frequency (RF) communication capabilities of smart devices carried by event attendees. Using Wi-Fi access points to scan for and count unique MAC-addresses is a viable solution and similar techniques can be utilized for Bluetooth communication as well. This does make the underlying assumption, however, that each attendee is carrying exactly one single smart device which has enabled Wi-Fi and/or Bluetooth communication. Furthermore, smartphone OS developers are increasingly implementing methods which repeatedly rotate through randomized MAC-addresses to directly foil the working principles of these types of scanning systems in order to enhance user privacy. While it is currently still feasible to identify unique devices from these semi-randomized MAC-addresses, the difficulty of doing so is likely to increase even more in the future [10]. A solution to this problem could be the use of a specific app instead of scanning for available devices, but this means that visitors need to be convinced to install this app on their device.

The use of passive RF sensing could provide a solution to all of these problems. The basic concept of this approach is to make use of the impact of the physical presence of a crowd on communication between RF-devices to infer information regarding the size and/or density of this crowd. Because the radio modules in the measurement devices are not only used for communication but act as sensors themselves, these type of approaches can also be called ‘sensorless sensing’. Sensorless sensing in general has become an increasingly popular research topic and techniques have been developed for a variety of applications such as device-free localization [11,12], activity recognition [13,14], health monitoring [15], gesture recognition [16] and fall detection [17]. A considerable amount of research has been performed regarding the use of sensorless sensing techniques for crowd counting as well [18,19,20]. Often, these approaches tend to solely make use of the impact of the crowd on signal strength measurements, but in the past few years several techniques have been developed which also incorporate Channel State Information (CSI) [21,22,23]. The increasing amount of commercially available Wi-Fi chips which allow easy access to CSI data is a major cause for its growing popularity within passive RF sensing.

Unfortunately, the vast majority of experiments which validated existing crowd counting approaches were performed in environments which simultaneously contained several dozens of people at most. To the best of our knowledge, the sole exception within the current state-of-the-art is the experiment we performed ourselves at the Freedom Stage of the Tomorrowland Music festival in 2017 [24]. In this real-life, commercial environment in which thousands of human individuals could be present, we installed 46 devices which each contained a 433 MHz and an 868 MHz transceiver. Over the course of three days, we collected data and then analyzed the evolution of the average Received Signal Strength (RSS) attenuation experienced within the RF network depending on crowd size and density. It should be noted that these terms were used interchangeably, as we only investigated the full crowd in the entire environment and did not look into the manner in which this crowd was distributed. Therefore, crowd density was regarded as a crowd count divided by the fixed environment size.

Because the experiment was performed in an uncontrolled environment, it was very difficult to obtain any kind of ground truth data regarding actual crowd sizes. Therefore, in our approach we made use of a set of low-quality camera images of the environment. We had these images manually analyzed by a team of volunteers who classified each image in one of six categories ranging from ‘0’ to ‘5’, with 0 representing a nearly empty environment and 5 being an environment that was nearly filled to the brim with people. These subjective human crowd analyses made it particularly clear how often the exact same visual information was interpreted in a vastly different manner by different people. The resulting smoothed out crowd categorizations were then assigned to the crowds at certain timestamps corresponding to the moments when the images were taken. While it could hardly be considered a reliable ground truth, this ‘visual validation’ data enabled us to investigate whether a passive RF-based system could perform at least as well as a large set of human eyes. We used the average attenuation of the 433 MHz and 868 MHz RF networks together with the corresponding visual validation classifications to train and evaluate a probabilistic neural network (PNN). Results indicated that it was indeed feasible to create an automatic crowd estimation system based on a passive RF sensor network, with over 90% of pnn estimates being at most 1 category removed from the visual estimates. This was still very much a preliminary feasibility study within a single environment, however. Therefore, we stressed that much more research was needed regarding the universal applicability of one such approach.

Over the past years, we have installed passive RF sensor networks in a variety of different large-scale event environments, some of which did enable us to have access to a much more solid type of ground truth data. In this paper, we will discuss several of these setups and show the analyses we performed upon the resulting RSS data. The environments in which these experiments took place are as follows:

- Freedom Stage (Tomorrowland 2017)
- Freedom Stage (Tomorrowland 2018)
- Main Comfort VIP Area (Tomorrowland 2018)
- Nucleus Tent (Sound of Science 2019)

The Main Comfort VIP Area could only be entered and exited by visitors who had their special VIP bracelet scanned and based on this scanning data, crowd estimates regarding the number of people present in the environment were calculated. While some aspects of the specific system setup caused the resulting people count to not be entirely accurate, it was still a much more reliable form of ground truth than what had previously been the case. For the Nucleus Tent environment, the setup we installed there was part of a controlled experiment we performed at a science festival with 247 participants. Therefore, we could manually verify the exact number of people present over the course of the experiment. Finally, for the Freedom Stage we were granted access to cashless payment data indicating the number of transactions at the drink stands within the area during each minute while the festival was ongoing. We received this data for both the 2017 and 2018 editions of Tomorrowland and therefore we will include the data from our initial experiment in [24] once again in our analyses.

It should be noted that we do no longer limit our analyses to a categorization of the entire crowd within the entire environment, as was the case in [24]. Instead, we will make use of the available ground truth and maximum environment capacity data in combination with a linear regression-based approach to perform actual crowd count estimations. Furthermore, we do not solely investigate the entire environment as a whole, but also delve briefly into the concept of subregions for the experiments performed within the Nucleus environment.

The remainder of this paper is structured as follows. In Section 2, we discuss the architecture of the passive RF sensor networks we installed in the aforementioned environments. It should be noted that sometimes considerable differences existed between these setups in regards to hardware, firmware, back-end and real-time visualization and we will therefore focus primarily on the general concepts behind our setup designs. The next three sections fully describe the experiments performed in the mentioned environment and discusses the obtained results. In Section 3 we perform extensive analyses of the Main Comfort environment for which we had access to reasonable ground truth data and could therefore assess the accuracy of an actual crowd counting system in a quantifiable manner. An analysis of the controlled crowd estimation experiment performed at Sound of Science 2019 is presented in Section 4. Section 5 focuses on the Freedom Stage environment in which no ground truth data was present and where we had to make use of cashless transaction data and expert opinion to gauge the feasibility and usefulness of our approach in a crowd management context. Finally, in Section 6 a conclusion is presented in addition to a discussion of our plans for future research.

## 2. Collecting RSS-Measurements in a Passive Radio Sensing Network

Over the past few years, the architecture of the passive radio sensing networks we install in our experimental environments has evolved considerably. Because our measurements tend to take place in real-life, commercial environments, many aspects need to be taken into account not just from a research perspective, but also from a practical point of view. Once an event is ongoing, it often becomes very difficult or even outright impossible to physically access the network nodes. Smooth, continuous operation needs to be guaranteed for the duration of the entire event and if any issues do crop up, we need to be able to solve them remotely.

As was the case in our earlier work [24], we solely make use of 433 MHz & 868 MHz within our sensing network. Our primary motivators for doing so are the increased range and penetration capabilities for (non-metallic) walls, objects and human individuals these sub-GHz frequencies offer, especially when compared to the more commonly used 2.4 GHz frequency band. The basic principle of the system is to make use of the impact of large-scale human presence on RF communication, but this impact may not be so great as to cause a significant decrease in the number of available links due to communication failures. For non-controlled, large-scale environments containing thousands of human individuals in which nodes are often installed behind walls, this is not an unrealistic scenario.

Additionally, we continue to limit ourselves to measuring the RSS values of the communication links. As mentioned in the previous section, CSI has become a popular signal descriptor within the research fields of RF based passive crowd estimation and passive RF sensing in general. However, the fact that CSI requires the use of OFDM-based Wi-Fi signals (2.4/5 GHz) precludes its use in our current implementation of the system. Furthermore, we believe that only using RSS-measurements will enable the use of extremely low-cost, dedicated hardware in actual commercial deployments. Nevertheless, we consider the use of 2.4 GHz and more informative signal descriptors for large-scale crowd estimation to be interesting topics for future study and will discuss our plans regarding these research directions in more detail in Section 6.

In this section, we will discuss the manner in which our networks operate to collect RSS measurements and describe several features we implemented to increase the flexibility of the system. Additionally, we will describe the different hardware nodes we constructed and made use of for the specific setups discussed in this paper. However, we will not go into detail regarding the specific differences between each setup and quantify the corresponding influence of these changes on the general stability of the network, as we consider this to be an entirely separate topic outside of the scope of this paper.

### 2.1. Node Hardware

**Figure 1:**
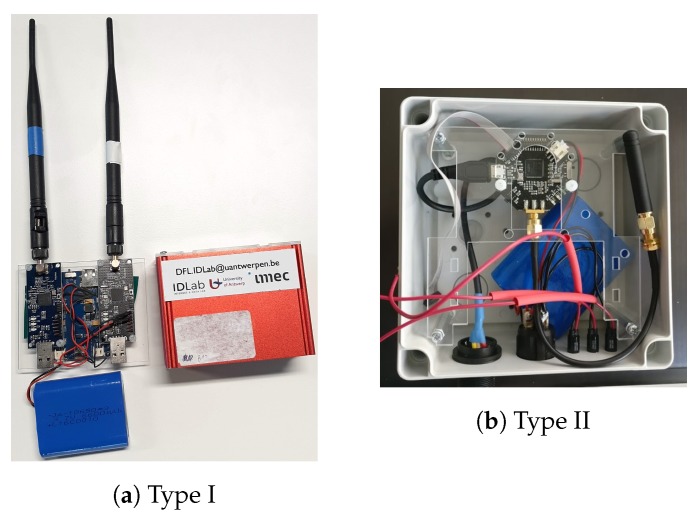
The two node types used for the experimental measurements. (**a**) shows an image of the main parts of which a Type I node consists and (**b**) shows an image of the inside of a Type II node variant containing only an 868 MHz transceiver.

Two different physical types of nodes were used for the environments which we discuss in this paper. Images of these nodes are shown in Figure 1. Our first node design consists of an aluminium, cuboid-shaped casing containing a battery (6600 mAh), a LiPo Rider Power module and two EZR-USB transceiver modules for respectively 433 MHz and 868 MHz communication. The casing is open on two sides, with one side covered by a plastic plate into which two holes were cut. These holes enable antennas to be connected directly to the modules. Because this design is not waterproof and has antennas which are clearly visible on the outside of the casing, it can only be used in indoor environments and is installed in places where it cannot be seen by event attendees. For the setups in this paper, this meant that nodes of this type were only installed in the Freedom environment in locations only accessible by crew members.

The design of the second node type is entirely closed with a casing made from plastic. It contains two RF transceiver modules which make use of an EZR microcontroller as well. Power is also supplied by a 6600 mAh battery, but this time the antennas are on the inside of the design. Additionally, the outside of the design contains a power switch, a USB-port for charging the battery and a set of green, yellow and red LED indicators. In practice these LEDs were not used. This node type was used for our setup in the Main Comfort environment and the Sound of Science controlled experiment.

### 2.2. Basic Network Operation

Communication between nodes within the network occurs through the DASH7 Alliance Protocol (D7AP) [25], an open standard sub-GHz wireless communication protocol which is generally used for medium distance Internet-of-Things (IoT) applications. Its open nature and utilization of license-exempt frequency bands (433 MHz & 868 MHz/915 MHz) make it ideally suited for our setups. While our sensorless sensing network differs from the more common sensor-actuator setups which form the primary use case for D7AP, there were nevertheless several beneficial DASH7-features which were quite helpful for our system. Later in this section, we will discuss this in more detail. Within our setups, there are three functionally different types of nodes: regular nodes, a controller node (controller) and a configuration node (configurator). The basic principle of communication within the network is that each in turn, the regular nodes broadcast a message which is then received by all other nodes. The RSS-value with which this message was received, is stored by each node in an internal list. This list is used as the actual data that is sent when a node’s turn to broadcast has arrived. The controller node listens to all of these messages as well, and it will pass on the RSS values they contain to a computing unit to which it is connected.

Regular nodes have unique node identifiers which determine the order in which they transmit. Their exact time to broadcast is synchronized based on an initial start message sent by the controller node. A node will broadcast its internal RSS list after *wtime* times the node identifier number of ticks have passed since the reception of this start message, with *wtime* being a network-wide parameter determining the time between the transmissions of two subsequent nodes. Once all nodes have transmitted their data, the controller node will broadcast another start message and the cycle begins anew.

It should be noted that the RSS data is received by the controller with a variable delay. The lists transmitted by regular nodes contain the RSS values with which it has received the most recent communications from each node, but these moments in time can differ significantly based on the network size and the the aforementioned *wtime* parameter. In practice, we consider these delays for our large-scale setups to be negligible as we make the assumption that the crowd does not significantly change within such short periods of time.

In addition to synchronizing node communication and passing the RSS data to a computing unit, the controller can also transmit several other types of messages to instruct the regular nodes to reset themselves or to request new network parameters from the configurator. The controller will send these messages if it receives the corresponding commands through its connection to the computing unit. These commands also include the option to respectively halt and resume the transmission of start messages.

The purpose of the configuration node is to ensure that all network parameters are known by all of the nodes. On boot, a node will start to regularly broadcast configuration request messages until it receives a response from the configurator. Only after this has occurred will the node listen to any start messages from the controller and participate in communication cycles. The network parameters received by a node include the aforementioned *wtime*, the size of the network and the unique node identifier. The file system-based nature of DASH7 makes this easily achievable by allowing us to store these parameters in user files on the configurator. Nodes can then obtain this data by transmitting a file read request. The only slight exception to this principle is the node identifier, which makes use of a table stored in the configurator. This table links each unique hardware ID of the transceiver modules to a node ID. When a read request is received, the hardware ID of the requesting node that is automatically included in each message will be used to determine the corresponding node ID which is then sent in response. Another highly important parameter is the access profile which defines a variety of communication-related parameters. In practice, it is primarily used within our system to define the frequency channel on which the regular cycle communication occurs. More in-depth information regarding the file system, access profiles and other general DASH7 concepts can be found in [25].

The main advantage the use of the configuration node provides, is the flexibility it offers in regards to the installation of our system. Increasing or decreasing the size of the network would require considerably more effort if these values were hard-coded in the firmware of each node. Additionally, we have previously encountered situations (e.g., during the Sound of Science 2019 measurements) in which already present signals within the environment interfered with the initially selected frequency channel for the main communication cycles. This could then be easily resolved by changing the corresponding parameters within the configuration node.

For the vast majority of the time, the network will be in its regular operation mode in which nodes synchronize their broadcasts based on the start messages sent by the controller. For this mode, the mean current drawn by the 433 MHz and 868 MHz transceiver modules combined will be below 35 mA for node type I 45 mA for node type II. Given that both node types contained a 6600 mAh battery, this meant that the corresponding battery run-times for types I and II were respectively 189 and 147 h. This was more than sufficient for the large-scale events described in this paper, which had a maximum duration of three days.

### 2.3. Back-End

RF transceivers which function as the controller and configuration nodes are installed within a gateway box and connected to a computing unit (in practice often an Intel NUC or a Raspberry Pi). This computing unit is then connected through the available network infrastructure to the actual back-end system, which is run on a local desktop/server or on a private cloud infrastructure. Communication between the gateway and the back-end occurs in both directions through the use of a topic-based message broker. All RSS messages received by the controller node are sent to the gateway computing unit and then in turn passed to the back-end through this system. Similarly, commands for the controller and network parameter changes for the configurator can be sent in the opposite direction. These are our main tools to address any issues that may arise while the system is operational during an ongoing event.

The back-end system itself has undergone significant changes since its inception and is still actively being developed. Initially, its main goal was simply to store the received RSS messages in a database from which they could be retrieved for analysis at a later date. Gradually, the system evolved towards an increased incorporation of real-time aspects. In particular, the evolution of the average RSS attenuation within (subregions of) the RF networks we installed was an important metric which we monitored through the use of interactive visualization platforms like Grafana [26]. Additionally, metrics related to the performance of the RF network itself (e.g., the ratio of expected RSS-messages received over the maximum within a cycle or specific window of time) were increasingly monitored as well to aid us in detecting problems more quickly.

## 3. Tomorrowland: Main Comfort 2018

The Main Comfort location at the Tomorrowland music festival comprises the ground floor of a temporary three-story building from which one has a clear view of the main stage. It can only be accessed by event attendees if they have VIP access and festival bracelets are scanned both when entering and exiting the environment. The two floors above the Main Comfort are respectively called the B2B & Skybox environment. A separate scanning system is in place for these locations because they require an even higher tier of VIP access to enter.

In theory, the Main Comfort scanning data provides us with an exact ground truth regarding the total amount of people present at any given time. In practice, however, there are several issues which negatively impact the accuracy of these numbers. First of all, crew members are not scanned and only have to show their bracelet to security personnel to be granted entrance. Second, part of the crowd that is present on the B2B and Skybox floors are included in the count as well. This is because it is possible to enter these environments by first going through the Main Comfort. Our system was only installed within the Main Comfort and not on the upper two floors and we did not have access to the data from their separate scanning system. Finally, bracelets were not scanned when people left the environment at the very end of the festival day, which meant that the crowd counts still remained unrealistically high after the day had officially ended.

Nevertheless, the measurements performed at the Main Comfort location in 2018 marked the first time that we had access to a representative crowd count for an environment which could contain thousands of human individuals. The results of our analysis, which we will discuss in the following paragraphs, strengthened our conviction regarding the validity of our passive RF sensing approach to create a full-fledged crowd analysis system.

**Figure 2:**
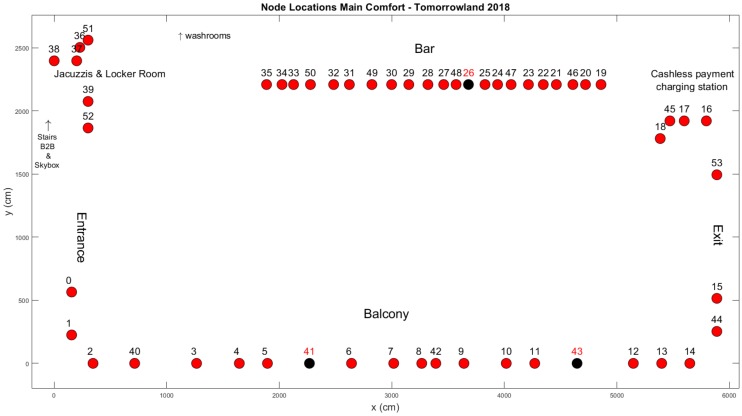
An overview of the Main Comfort environment.

In Figure 2 a schematic overview of the environment is shown, with the locations of the regular nodes we installed marked with dots alongside the corresponding node ids. Our setup consisted of 54 nodes of the type shown in Figure 1b. During operation when the festival was ongoing, however, we noticed that data was not received from three nodes due to a variety of hardware issues. This meant that the actual total number of functional nodes in this environment was 51. The three faulty nodes are indicated in Figure 2 with black dots and red node IDs.

The locations in the environment where we installed our nodes were primarily selected based on practical considerations. While it was not considered to be a problem for the type II nodes installed in the Main Comfort environment to be seen by event attendees, for obvious reasons their presence had to be as non-intrusive as possible. With the exception of node 53—which was installed right below a wooden shelf close to the exit—none of the nodes could be physically accessed by regular crowd members. The nodes on the balcony (0–15 & 40–44) were tied to its outermost guardrail, which was only accessible to security personnel. The nodes near the jacuzzis and the locker room (36–39 & 51–52) were hidden behind wooden walls and the remaining nodes were placed underneath the wooden bars for both the food & drink stand (29–35 & 46–50) and the cashless payment charging station (16–18 & 45). Due to these considerations and the fact that elevation differences existed between (parts of) the balcony and the rest of the environment, nodes could not all be installed at the exact same height. Therefore, the Lines-of-Sight of the communication links should not be considered to be parallel to a uniform ground plane. Nevertheless, we attempted to install the nodes at heights between 1 m and 1.60 m to ensure that the direct line-of-sight of most links went through the torsos of adult individuals standing upright. For the vast majority of cases, this was feasible.

**Figure 3:**
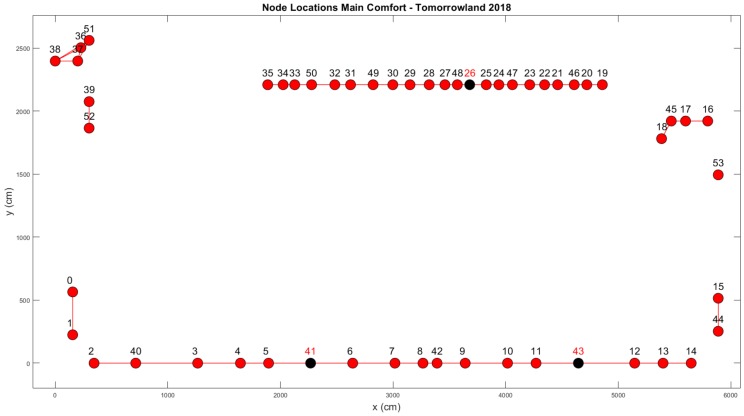
An overview of the discarded links within the Main Comfort environment.

The installation of 51 functional nodes led to a total of 1275 two-way communication links which provide us with two RSS measurements each for every successful cycle. Because we are interested in using the influence of the crowd on the RSS values of the communication links within the environment to perform crowd estimation and crowd analysis, we will not use data from links which are unlikely to be impacted. These links are indicated in Figure 3 and their measurements are not taken into account during the next steps. In practice, this meant that only 895 links were used.

### 3.1. Average RSS Attenuation & Calibration

Our earlier research performed in [24] indicated that the average RSS attenuation experienced within an installed RF network was a solid indicator of the actual crowd size. Now that we have access to the bracelet scan data for the Main Comfort environment and our own manual counts for the Sound of Science environment, we will investigate the use of this metric more thoroughly.

We define average RSS attenuation as the average of the differences between our current link measurements and an earlier set of calibration measurements which were performed when the environment was (largely) unoccupied. In practice, rather than calculating the average RSS attenuation for each cycle, the RSS difference values of every communication link are averaged within a certain time window and a single attenuation value is calculated for every window. For the Main Comfort environment analyses described in this section, this time window had a length of 60 s.

Calibration measurements are performed during a relatively short period of time (which can range from 5 min to 1 h) when the environment is largely static and empty. The average RSS measurements for each link obtained during this period represent an unoccupied environment. If any significant changes take place regarding the generally static elements within the environment (e.g., the locations of furniture or the installation of additional crowd barriers), new calibration measurements need to be performed. In order to mitigate the problem of small environmental changes accumulating over time, we use a different set of calibration measurements for each day of the festival. The extent to which changes must occur in an environment for a new calibration measurement to be necessary, is currently unknown. Because calibration measurements require an unoccupied environment, they are impossible to perform while the event is actually ongoing. This means that large environmental changes while an event is ongoing can have a significant negative impact on the crowd estimation accuracy of the system. Therefore, this is an important topic for future research which we will discuss in more detail in Section 6.

**Figure 4:**
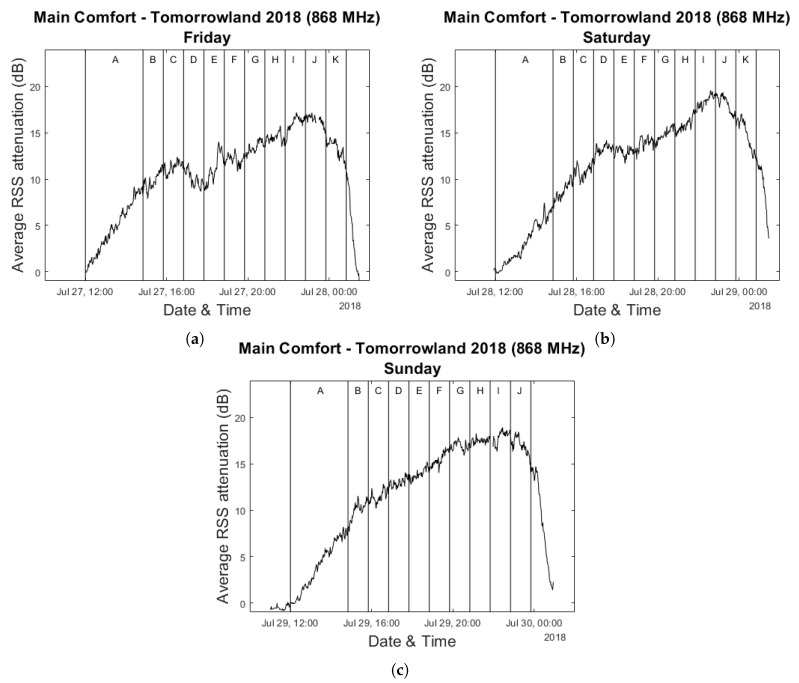
Evolution of the average RSS attenuation within the Main Comfort Environment throughout the festival weekend. (**a**–**c**) show this for respectively Friday 27 July 2018, Saturday 28 July 2018 and Sunday 29 July 2018.

Figure 4 shows the evolution of the average RSS attenuation during all three days of the festival for 868 MHz. The vertical lines divide the duration of each day into time slots based on the performances on stage, with each letter indicating a different artist for the day. Measurements performed during the first five minutes after the opening of the festival grounds at 12:00—when preparations were certain to have been completed but when the crowds had not yet arrived at the Main Comfort—were used as calibration data for each day. We will primarily focus on Saturday and Sunday due to the fact that the available bracelet scanning data for Friday was largely incomplete.

While we performed measurements on both the 433 MHz and 868 MHz frequency bands, in this paper we only make use of the 868 MHz data. The results of our earlier study described in [24] already indicated that the resulting data from these two frequencies for large crowds were very strongly correlated (433 MHz & 868 MHz essentially show the same graph with an offset) and was unlikely to provide any additional information.

### 3.2. Bracelet Scanning Data

**Figure 5:**
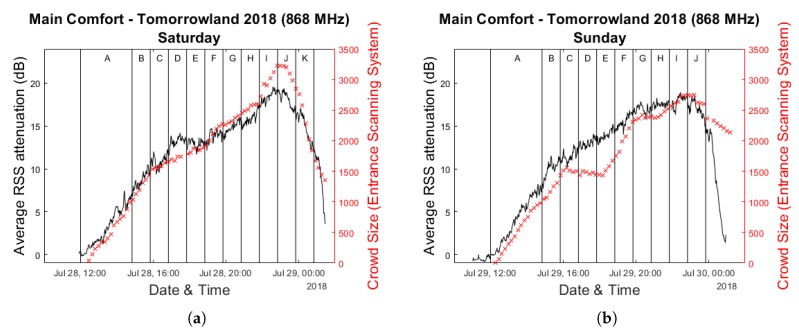
Evolution of the average RSS attenuation within the Main Comfort Environment, overlaid with the scanning system data. (**a**,**b**) show this for respectively Saturday 28 July 2018 and Sunday 29 July 2018.

In Figure 5 the same graphs as in Figure 4 for Saturday and Sunday are overlaid with the crowd estimates provided by the bracelet scanning system. The estimate of the scanning system was updated approximately every 10–12 min, which contrasts with our own approach which—for the window size of 60 s we have selected—provides a new average RSS attenuation value every minute. For this reason, when comparing these two sets of data we will always compare each scanning system value to the corresponding attenuation value that is closest in time. Additionally, we completely disregard any data which was collected after the last performance of the day had concluded due to the previously mentioned lack of people scanning at the end of the day.

**Figure 6:**
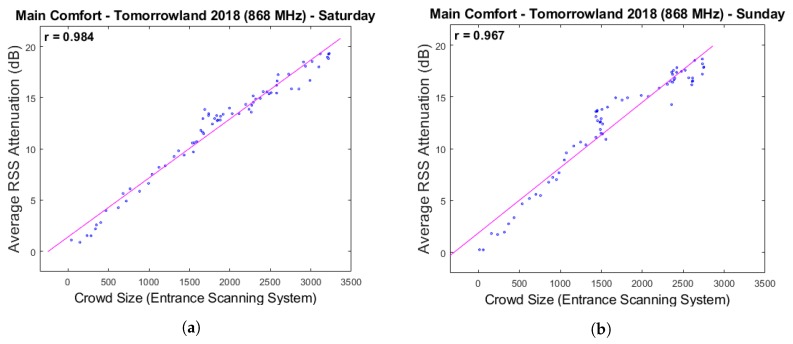

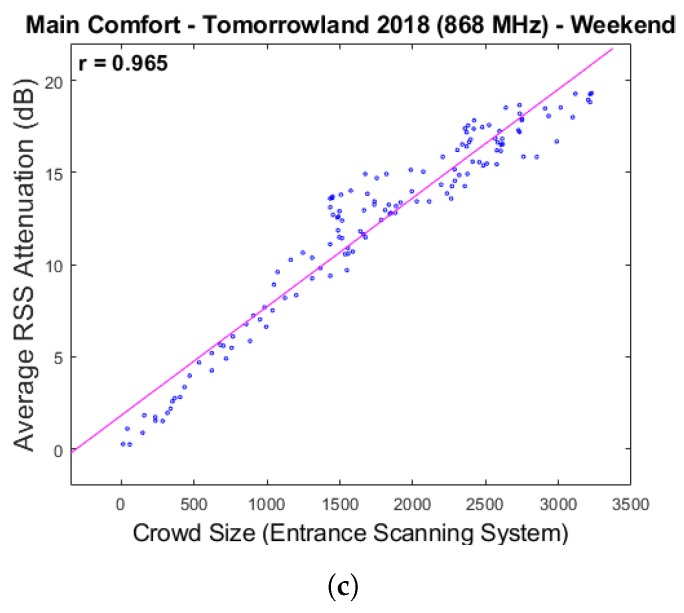
Scatter plots depicting the relationship between the average RSS attenuation and the crowd counts according to the scanning system in the Main Comfort environment. Plots (**a**,**b**) make use of the data from respectively Saturday 28 July 2018 and Sunday 29 July 2018, while plot (**c**) uses all available data from both days combined.

Visually speaking, it is already quite clear from Figure 5 that strong correlations exist between the two data sets. The scatter plots in Figure 6 show very high Pearson correlation coefficients of respectively 0.984, 0.967 and 0.965 for Saturday, Sunday and the two days combined. These results strengthen our hypothesis that the average RSS attenuation within an RF network is a strong indicator of the crowd size and can therefore be used as a solid basis for a passive crowd estimation system. It should be noted, however, that there are some time intervals in which these correlations appear to be much less strong and even very slightly negative. This is particularly visible in the comparative graph for Sunday, which shows a decrease in the number of people present according to the scanning system for approximately 2 h after 16:00u. During the same period of time, the average RSS attenuation increases steadily, albeit at a slower rate than before. Much less pronounced but similar behaviour can be observed for Saturday within the same time frame. Because this occurred at approximately the same time when complimentary food and drinks were starting to be served within the environments above the Main Comfort, we suspect that this discrepancy could be related to the fact that the upper floors are partially included in the scan counts as well. People who increasingly make use of the direct entrance to the upper environments without first passing through the Main Comfort could be a potential explanation. However, with the currently available data, this is impossible to verify.

### 3.3. Estimating Crowd Sizes

**Figure 7:**
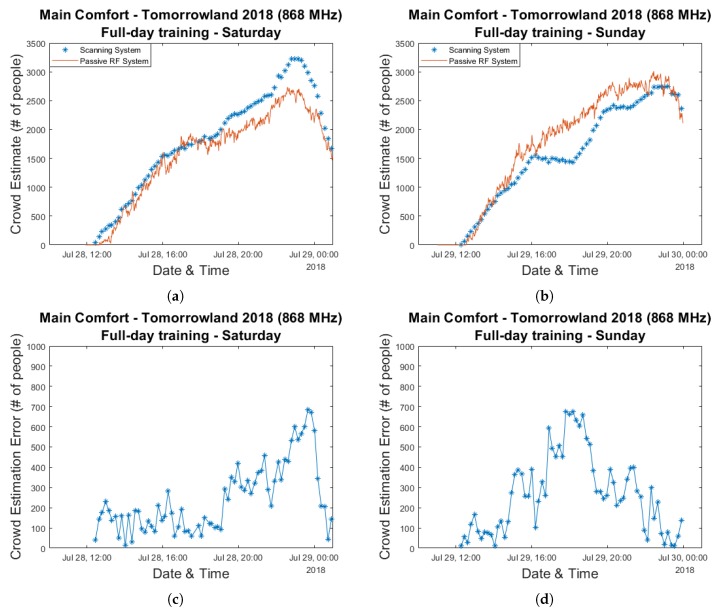
Crowd estimation graphs and their corresponding error graphs for a model trained using the data from an entire day and evaluated based on the data collected during the other day. Plots (**a**,**c**) were created based on training data collected on Sunday 29 July 2018 and evaluated based on data from Saturday 28 July 2018. Plots (**b**,**d**) were created based on training data collected on Saturday 28 July 2018 and evaluated based on data from Sunday 29 July 2018.

Now that the average RSS attenuation has proven to be a strong indicator for crowd size, our next step consists of the creation of a model which translates this value into actual crowd estimates. We make use of a linear regression-based approach to determine the equation describing the RSS attenuation-crowd size relationship. We investigate three different methods for selecting which data is to be used for training and which data is to be used used for evaluation. In our first approach, we simply utilize the curves depicted in Figure 6a,b which are fitted for all data points from a single day and evaluate the accuracy using the data from the other day. Figure 7 shows the resulting estimation graphs and their corresponding error graphs for Saturday and Sunday.

Maximum errors close to 700 people can be seen for both days. Interestingly, on Sunday this occurs during the 16:00–18:00 period which we discussed previously while for Saturday this peak is reached near the end of the festival day when the crowd size was at its largest. If one compares the graphs in Figure 5a,b, a significant underestimation of the maximum crowd count on Saturday based on a curve fitted to data from the following day is entirely unsurprising and we will see this occur for all three of our approaches. This is because the maximum values for the average RSS attenuation are comparable for both days (19.6 dB and 19.0 dB), while according to the scanning system the largest amount of people who were simultaneously present within the environment differs considerably. Once again, it is very difficult to assess whether this was caused by inaccuracies related to the scanning approach or if environmental or crowd-related factors caused a different relationship between the crowd size and the average RSS attenuation within our RF network.

**Figure 8:**
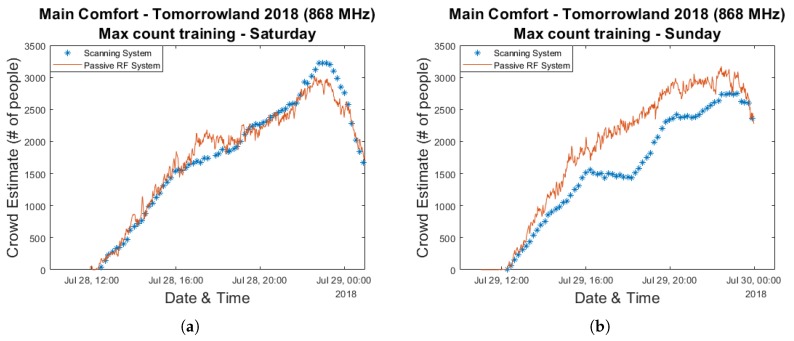

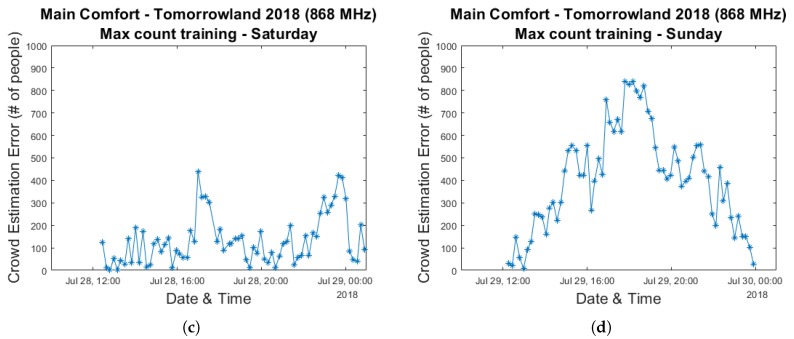
Crowd estimation graphs and their corresponding error graphs for a model trained using the max count from a single day and evaluated based on data collected during the other day. Plots (**a**,**c**) show the results of training based on the max count of Sunday 29 July 2018 and evaluation based on data from Saturday 28 July 2018. Plots (**b**,**d**) show the results of training based on the max count of Saturday 28 July 2018 and evaluation based on data from Sunday 29 July 2018.

For our second approach we select the maximum count according to the scanning system for each day. The equation of the curve which connects the origin to this value and its corresponding RSS attenuation are then used as our model and data from the other day is used for evaluation. The resulting graphs are shown in Figure 8. This leads to a maximum crowd estimation error below 450 people for Saturday, but for Sunday the actual crowd count according to the scanning system was consistently overestimated.

**Figure 9:**
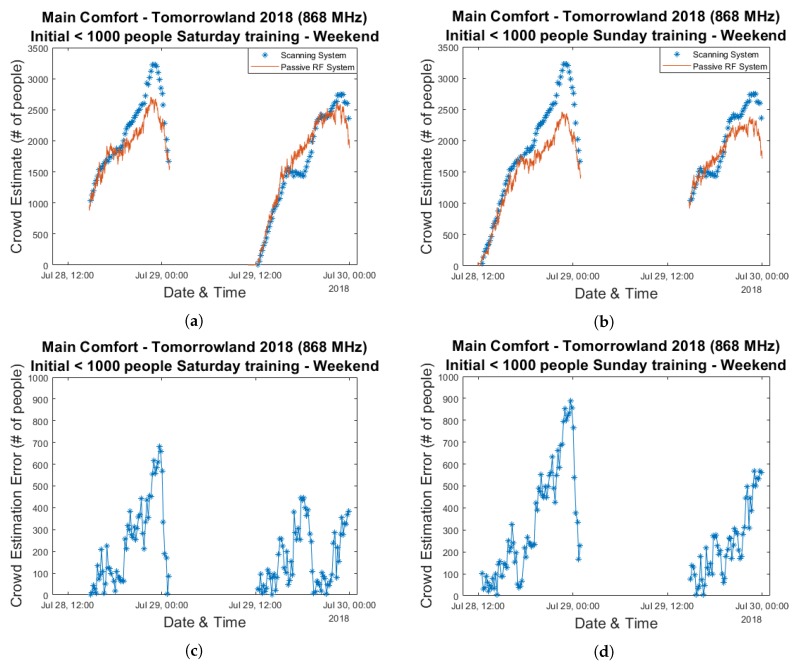
Crowd estimation graphs and their corresponding error graphs for a model trained using the data from a single day up until a crowd size of 1000 people was measured by the scanning system and evaluated based on all remaining data collected during both days. Plots (**a**,**c**) were created based on training data collected on Saturday 28 July 2018 and evaluated based on the remaining data from both days. Plots (**b**,**d**) were created based on training data collected on Sunday 29 July 2018 and evaluated based on the remaining data from both days.

Finally, for our third approach our training set consists of the data from the beginning of the day up until the scanning system indicates more than 1000 people are present. All other data—including from the same day—is used for evaluation. This is the type of method one would use in an actual real-life crowd estimation system in which initial crowd counts can still be performed manually or potentially in combination with a camera-based setup and the resulting data is used to train the RF-based passive sensing system. Results are shown in Figure 9 and they indicate decently accurate results for Sunday while the underestimation of the peak period on Saturday is still quite severe.

**Figure 10:**
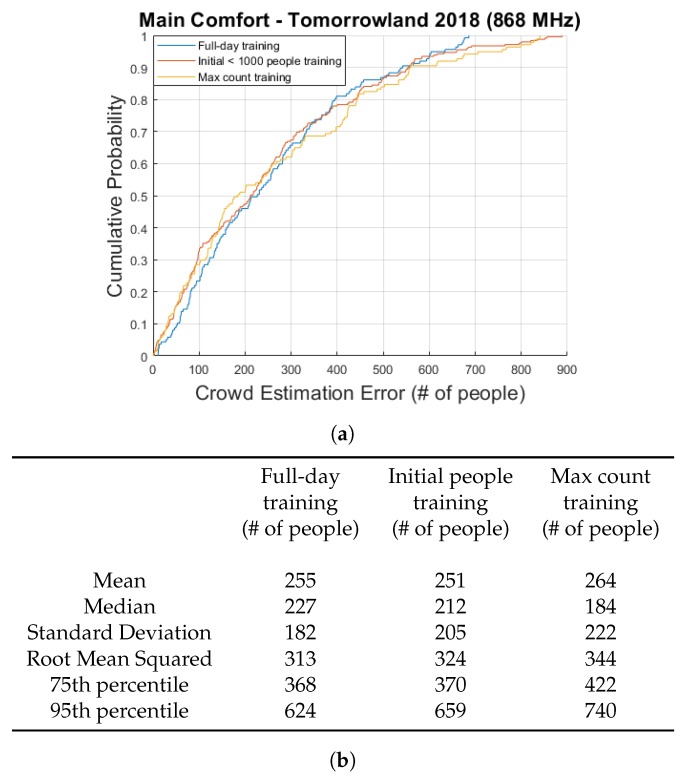
A cumulative distribution function plot comparing the obtained crowd estimation error values for the Main Comfort data for three different training approaches. (**a**) shows the actual plot while the table shown in (**b**) contains several key statistical metrics.

Figure 10a shows a graph of the cumulative distribution functions of all error values for the three approaches. Median errors of respectively 227, 212 and 184 people (7.0%, 6.6% and 5.7% of the maximum count of 3227) were obtained, which we consider to be quite impressive for straightforward linear models. Standard deviations are quite large, however, and the lowest 95th percentile is equal to 624 people. We have previously identified two main periods of time during which the errors became excessively large: 16:00–18:00 when the complimentary food and drinks began to be served in the environments above the Main Comfort and the peak moments near the end of the day when the crowd size was at its maximum. These large discrepancies were shown to likely be the result of the fact that the largest mean RSS differences were quite similar for both days while the crowd scanning system estimated a significant difference of nearly 500 people between the maximum crowd sizes. When data from one day is used as training and the other for evaluation, the results are predictable. While the obtained 95th percentile error values would be problematic for a fully implemented, commercial crowd estimation system, it should nevertheless be kept in mind that the accuracy of the scanning system is very much in doubt and that the festival organizers considered our estimates to be feasible and useful. However, it is quite clear that further research is necessary.

**Figure 11:**
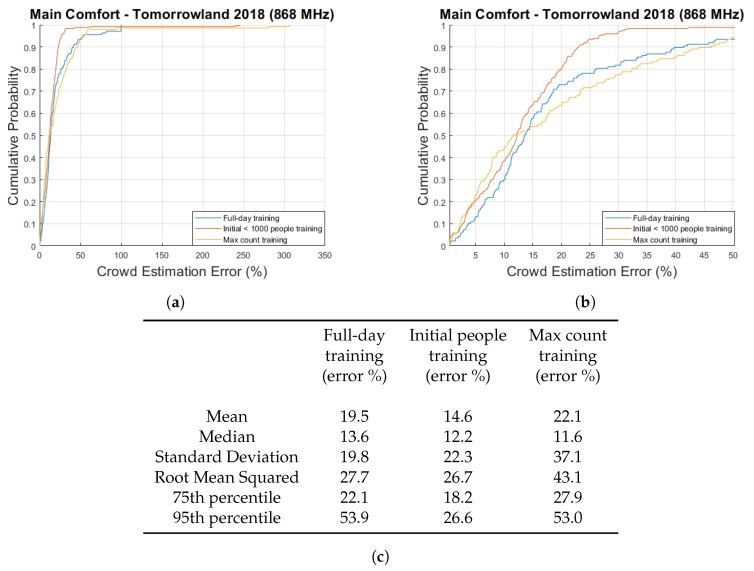
A cumulative distribution function plot comparing the obtained crowd estimation error value percentages for the Main Comfort data for three different training approaches. (**b**) zooms in on a portion of (**a**) and the table shown in (**c**) contains several key statistical parameters.

The reason we calculated the previously mentioned percentages based on the maximum count, is because this is usually the value that event organizers are most interested in in the context of crowd safety. Another possible approach to represent these errors in a percentage-wise manner, is to simply calculate each error value as a percentage of the current crowd size according to the scanning system. To give an example, crowd size estimates of 90 and 110 would both give an error percentage of 10% if the true crowd count according to the scanning system were 100. The cumulative distribution function plots showing the results of this approach are shown in Figure 11a,b.

**Figure 12:**
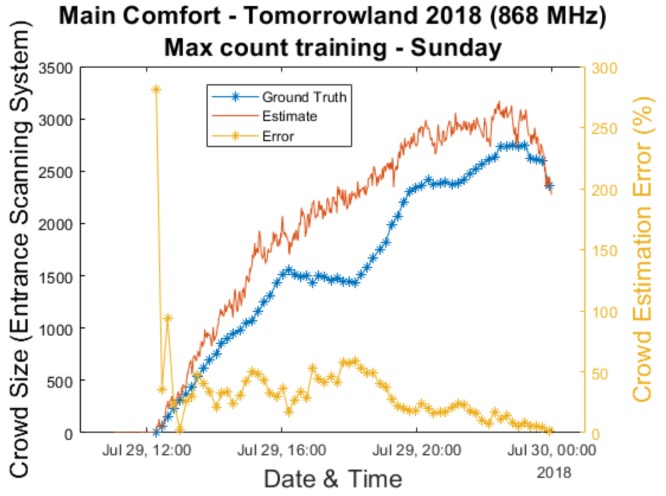
A graph showing the evolution of the crowd estimate of the passive RF based system versus the estimate of the scanning system and the resulting error percentage between the two for the final festival day.

Figure 11a zooms in on Figure 11b to give a clearer overview of the error percentages up to 50%. This is necessary due to outliers which tend to occur during the early moments when the crowd count (according to the scanning system) is still very low. This is clearly illustrated by Figure 12, in which it can be seen that the estimation error percentage briefly lies between 250% and 300% during the beginning of the final festival day when using the max count training. The cumulative distribution function plots seemingly show a result that is particularly positive for the initial people training method. This result is not surprising, given the fact that we showed earlier that the largest errors for this approach occurred near the end of the festival when the crowd count was high. Once again, we must point out that it is still unclear as to whether this consistent underestimation is primarily related to the previously mentioned issues with the scanning system or due to inaccuracies regarding our own approach.

## 4. Sound of Science: Nucleus

Sound of Science is a Belgian popular science event which aims to introduce scientific concepts to a general audience. During its 2019 edition, we were granted the opportunity to perform a controlled crowd estimation experiment in which event attendees could participate. It took place in the ’Nucleus’ tent environment of approximately 530 $m^{2}$ in which we installed 44 nodes. Data was only captured on the 868 MHz frequency band and the total amount of volunteers was equal to 247. The full experiment consisted of 16 sub-experiments during which the participants were asked to perform a certain action (raise their hands or sit down/stand up) or for a specific number of them to leave the environment. This was accomplished by handing out sheets of paper containing an experiment number, with participants being asked to leave when their experiment number was called out. On exiting the environment, they were required to hand over their sheet which allowed us to detect cases in which people with an incorrect experiment number still left the environment and update our ground truth counts accordingly. Additionally, a consumer-grade camera device was installed as well and therefore we had access to low-quality images of the environment while the experiments were ongoing, although these do not provide a full overview of the entire environment.

Given the focus of this paper on crowd estimation, in our analysis we will primarily look at the sub-experiments in which people left the environment. This was the case for experiments 6 to 16, with the sole exception of sub-experiments 9 and 10 during which the participants were asked to respectively sit down and stand up again. In our analysis graphs within this section, the experiments are labeled A (sub-experiment 6) to K (sub-experiment 16).

**Figure 13:**
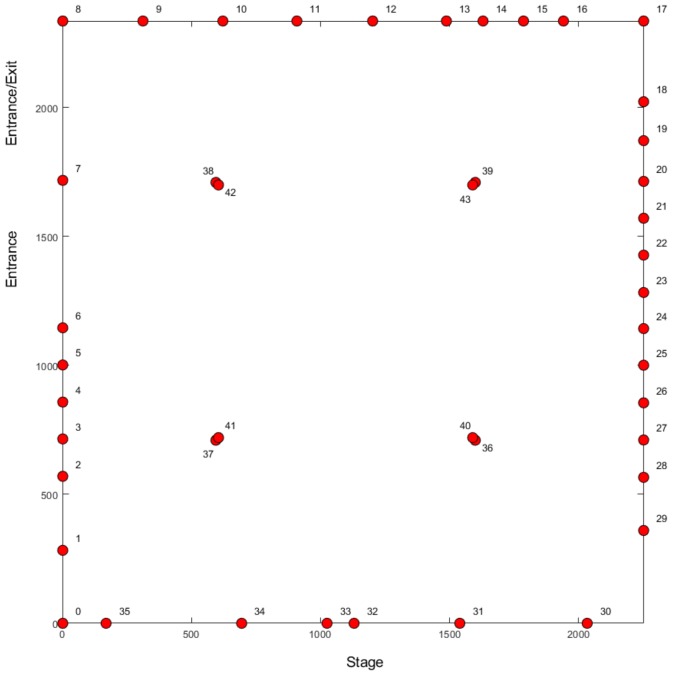
An overview of the Nucleus tent environment.

**Figure 14:**
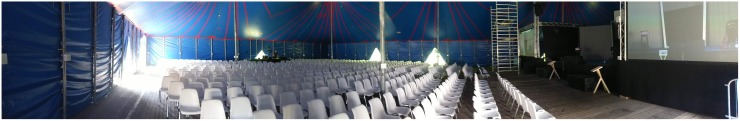
A photograph of the Nucleus tent environment.

A schematic overview and a photograph of the environment are respectively provided in Figure 13 and Figure 14. Nodes were attached to metallic support poles both at the edges of and within the tent environment. Due to the controlled nature of the experiment and the scientific theme of the event, it was not considered to a problem for participants to have physical access to them. They were installed at approximately 1.50 m from the ground, which was near the upper end of the range of installation heights for the nodes in the Main Comfort environment. The main reason for this increased height was the fact that—in addition to regular crowd size estimation—we also wished to investigate the feasibility of differentiating between a crowd whose members are standing up versus a crowd whose members are sitting down. This is also the reason why sub-experiments 9 and 10 were included in the controlled experiment. As a research topic we consider this to be out of scope for this paper, but we are planning on investigating this in future. We will discuss this in more detail in Section 6.

The use of 44 nodes resulted in a total of 946 communication links. As was the case for the Main Comfort environment, we disregard the data from communication links whose lines-of-sight do not intersect the environment in which the crowd is present. In practice, this means the links between the nodes surrounding the environment whose locations form a set of collinear points, with the exception of the link between nodes 6 and 7 and the link between nodes 7 and 8 due to their presence near the entrance and exit. Therefore, only 755 links were used.

### 4.1. Average RSS Attenuation & Ground Truth

As we have done for the Main Comfort environment, we analyze use of the average RSS difference metric for crowd estimation. Calibration measurements were performed during a short 5 minute period approximately 20 min before the experiments were announced to begin. Because the full experiment was performed over a very short period of time (approximately 12 min), the window size is drastically reduced compared to the Main Comfort analysis in order to have sufficient data points. As a result, a new average RSS attenuation value is calculated based on all data collected during every 5 s.

**Figure 15:**
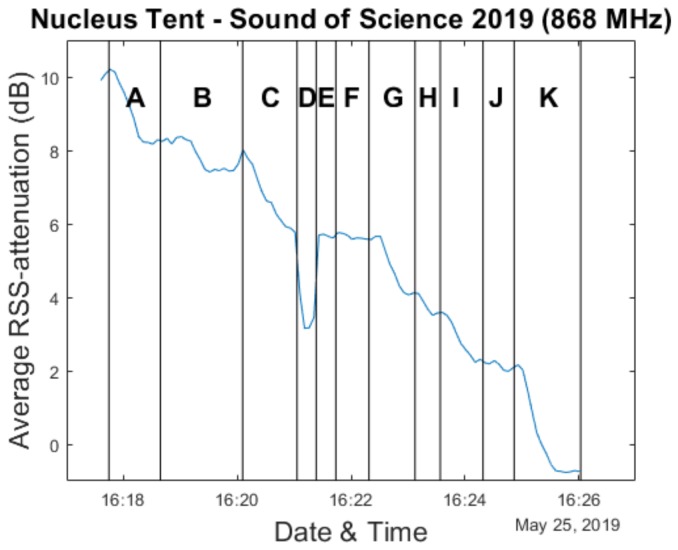
Evolution of the average RSS attenuation during the experiments in the Nucleus tent environment.

**Figure 16:**
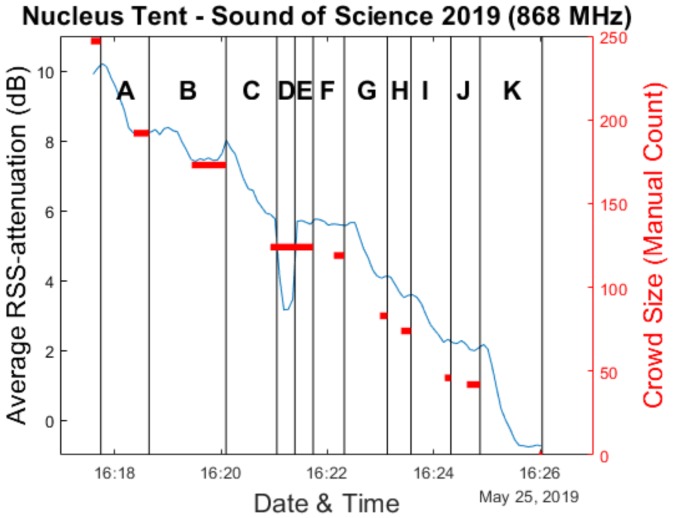
Evolution of the average RSS attenuation during the experiments in the Nucleus tent environment, overlaid with the ground truth data.

Figure 15 shows the resulting evolution of the average RSS attenuation compared to our manually obtained ground truth. The exact number of participants still present in the crowd could only be known during the short periods of time at the end of an experiment when no participants were still in the process of exiting the environment. Special attention was paid to delineate these periods while the experiments were ongoing. As a result, the graph in Figure 16 only shows a ground truth value for these moments. This also means that when comparing our measurements to the ground truth, we only make use of the average RSS attenuation values which were determined during these time intervals. Furthermore, the data from experiments D and E are discarded as well, as the changes in average RSS attenuation are caused by the participants sitting down and standing back up again, rather than by changes in crowd size. In practice, this leads to there being only 25 data points available for the analyses we perform.

**Figure 17:**
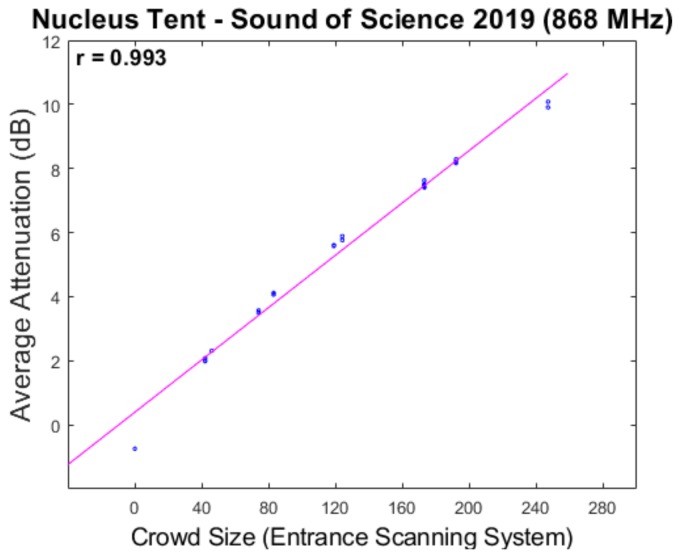
Scatter plots depicting the relationship between the average RSS attenuation and the ground truth in the Nucleus tent environment.

A scatter plot and the associated Pearson correlation coefficient is shown in Figure 17, indicating an extremely high correlation between our measurement value and the ground truth. Once again the feasibility of our passive RF sensing approach is shown, this time in an environment with significantly lower crowd sizes compared to our earlier experiments.

### 4.2. Estimating Crowd Sizes

**Figure 18:**
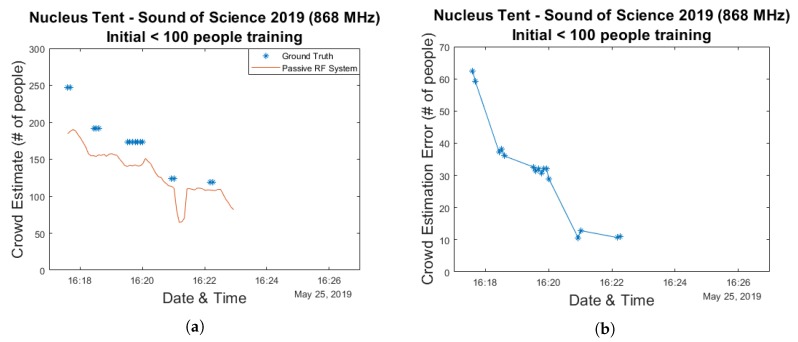
A crowd estimation graph and its corresponding error graph for a model trained using the data from the Sound of Science experiments for which the actual number of people present was below 100 and evaluated based on all remaining data. (**a**) shows the crowd estimation graph and (**b**) shows the error graph.

**Figure 19:**
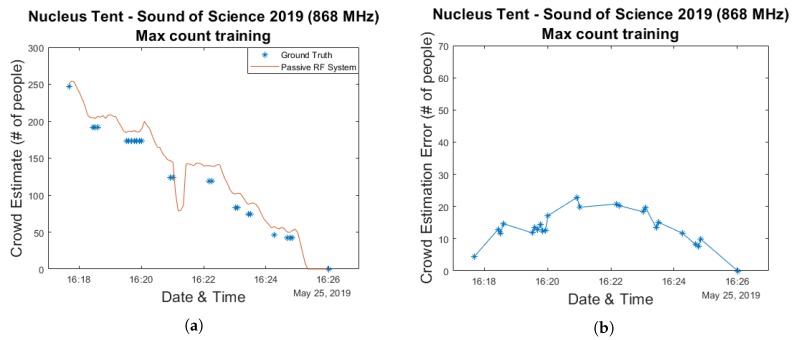
A Crowd estimation graph and its corresponding error graph for a model trained using the max count from the Sound of Science experiments and evaluated based on all remaining data. (**a**) shows the crowd estimation graph and (**b**) shows the error graph.

**Figure 20:**
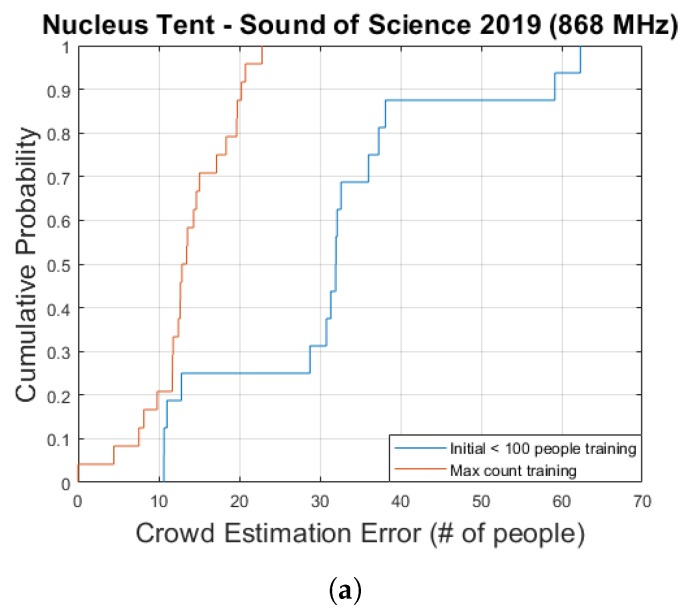

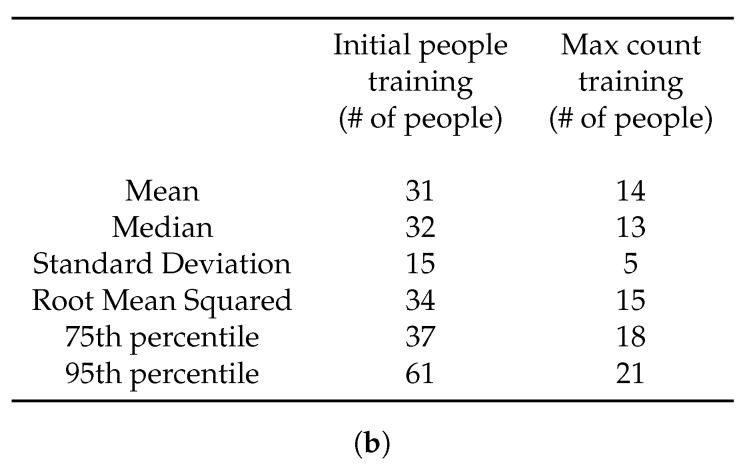
A cumulative distribution function plot comparing the obtained crowd estimation error values for the Nucleus tent data for two different training approaches. (**a**) shows the actual plot while the table shown in (**b**) contains several key statistical metrics.

As was the case for the Main Comfort environment, we investigate the use of a linear regression-based approach to perform actual crowd estimations in this environment as well. Both the training approach which simply makes use of the maximum crowd count as well as the approach in which the model curve is fitted to the data collected while the total crowd size was less than 100 are used. The resulting estimation and error graphs are shown in Figure 18 and Figure 19, while Figure 20a contains the corresponding cumulative distribution function plots. When interpreting these results, it should once again be kept in mind that the number of available data points is very limited. For the second method this means that 9 data points are used for training while the remaining 16 are used for evaluation.

As can be seen in the graphs the resulting median errors are quite low—13 & 32 people for the max count and initial training approaches, respectively. This corresponds to 5.3% and 13% of the maximum number of people present in the environment. The initial training based method significantly underestimated the actual crowd count at its peak, however.

### 4.3. Crowd Size Estimation within Subregions

**Figure 21:**
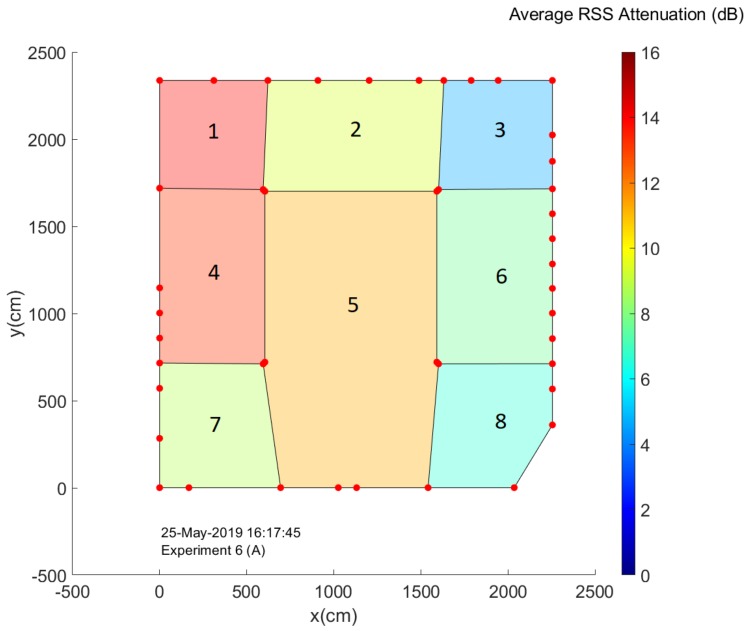
An overview of the manner in which the Nucleus tent environment was divided into 8 subregions. The color of each subregion represents the average RSS attenuation experienced by communication links within this region at the indicated date and time.

When one wishes to construct an actual crowd analysis system to aid in crowd management for event organizers, it is often insufficient to only have access to estimates of the total amount of people present in the environment. Information regarding the manner in which the crowd is dispersed throughout the environment can be crucial as well. After all, dangerous situations can still occur in environments with a low average number of people per square meter if the vast majority of this crowd is located within a small part of the total environment. For this reason we investigate the concept of subregions or zones, in which we divide the environment into multiple slices based on the locations of our nodes. A set of links is assigned to each subregion and average RSS attenuation values are calculated based solely on the data from these links. These values are then considered to be representative for the corresponding zone. Figure 21 shows the manner in which we divided the Nucleus tent environment into 8 different subregions. It should be noted that this division was performed in a somewhat arbitrary manner and it is chiefly meant to illustrate the feasibility of the concept. Our main concerns when choosing the subregions in which to divide the environment were simply to ensure that each subsection had sufficient nodes and that the regions could potentially be useful to analyze the movement of the crowd when leaving the environment.

**Figure 22:**
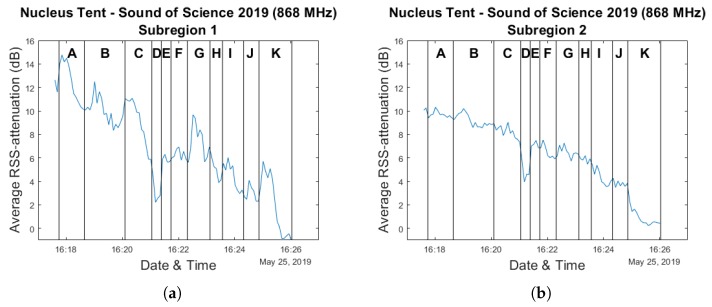

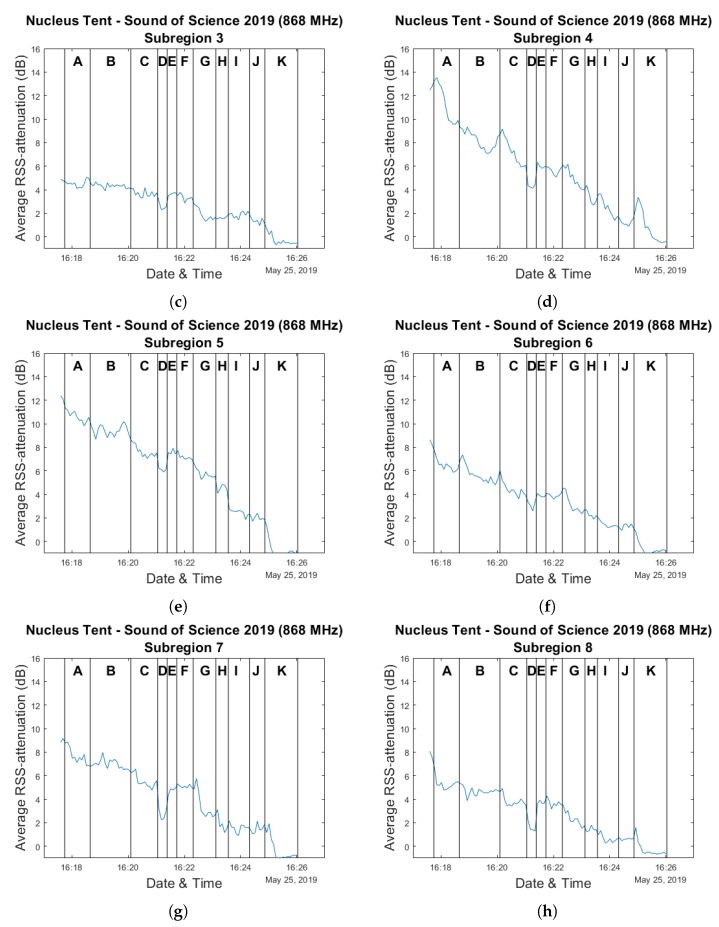
A set of graphs showing the evolution of the average RSS attenuation experienced within each subregion over the course of the experiments performed within the Nucleus tent environment. Plots (**a**–**h**) correspond to subregions 1 to 8.

Figure 22 shows the separate evolution of the average RSS attenuation for each zone over the course of the experiments. Based on these graphs, several patterns can be quickly identified. Unsurprisingly, given the fact that subregion 1 is located near the environment exit, rapid increases followed by decreases can be observed during the course of almost every experiment. Furthermore, it is clear that the attenuation values for the zones on the right side of the environment (3, 6 and 8) are much smaller compared to the other subregions.

**Figure 23:**
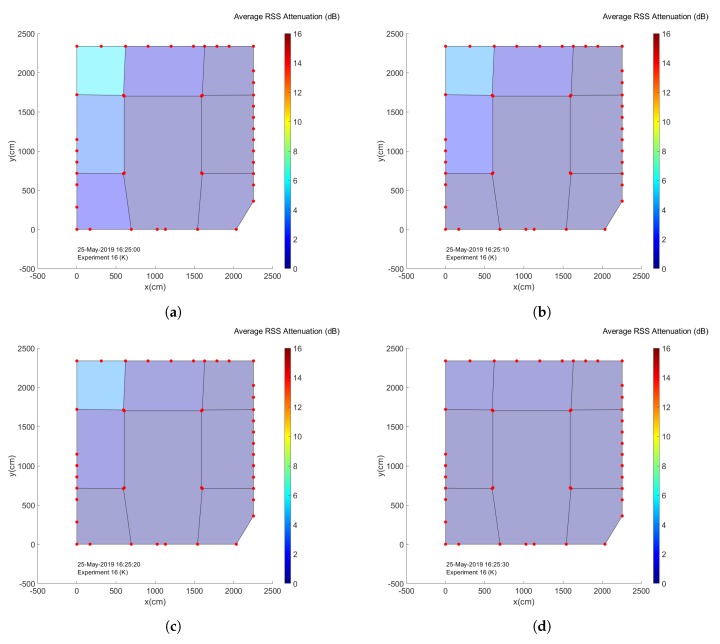
A set of attenuation maps providing an overview of the average RSS attenuation within each subregion near the end of the experiments, from (**a**–**d**) 16:25:00–16:25:30.

An interesting way to visualize all of these graphs, is by creating environment maps in which the colour of each subregion is determined based on its current average attenuation value. Representing the data in this manner makes it quite clear that the concept of subregions can be used to determine crowd flows within an environment. In Figure 23, this is illustrated by a set of four environment maps corresponding to the end of the experiments when the last groups of people were leaving the environment through the exit near subregion 1. An animation showing the evolution of these maps for the entire experiment, next to a corresponding series of low-quality camera images of the environment at the time can be found within the Supplementary Materials of this paper.

The approach we have taken when analyzing subregions is the same as for the entire environment: simply calculate the mean RSS difference of all the measurements from the selected links. While the results thus far have shown this to be a valid methodology for our large-scale environments, it is unclear how strongly this holds true for subregions. No distinction is made between different links regarding the information they provide, even though they can be impacted in drastically different ways by human presence and movement due to differing node locations, node distances and a variety of multipath effects. While this is a remark which can be made regarding our approach in general, the smaller amount of links in a much smaller subregion environment makes a coarse approach in which a non-weighted mean RSS difference value is assumed to be linearly correlated with the crowd count much less likely to be effective. If one looks at existing small-scale crowd estimation (or general device-free localization) techniques, many approaches already take this into account. Examples of this include the concept of link fade level in Radio Tomographic Imaging (RTI) [27,28,29] and the explicit non-linearity of Sequential Counting, Parallel Localizing (SCPL) [30]. Combining fine-tuned approaches for specific small-scale subregions—a domain for which substantial research already exists as previously mentioned in the introduction—with a more coarse for the greater environment could be a very important future research direction.

## 5. Tomorrowland: Freedom Stage 2017–2018

It was in the Tomorrowland 2017 Freedom Stage environment where we performed our first large-scale passive RF sensing measurements which were actively used for a crowd estimation study as described in [24]. In 2018, we repeated our experimental measurements within the same environment. For these measurements, the lack of ground truth data was even more egregious than the year before as we no longer had access to low-quality camera footage of the environment. After this event, however, the festival organizers permitted us access to the cashless transaction data for the Freedom Stage. Within the entire grounds of Tomorrowland, all payments occur in a cashless manner by scanning the buyer’s festival bracelet. The data provided to us gives an overview of the total amount of cashless transactions that have taken place within the Freedom Stage during each minute for both the 2017 and 2018 editions of the festival. Based on expert opinion and our personal notions of the manner in which the crowd size evolved throughout each festival day, we assume that this data is positively correlated with the actual number of people present in the environment. Therefore, in this section we will investigate the correlations between our average RSS attenuation metric and the cashless transaction data. While it would not be conclusive in and by itself, the presence of a clear correlation could be considered to be yet another piece of evidence strengthening the validity of our passive RF sensing based approach to crowd estimation.

**Figure 24:**
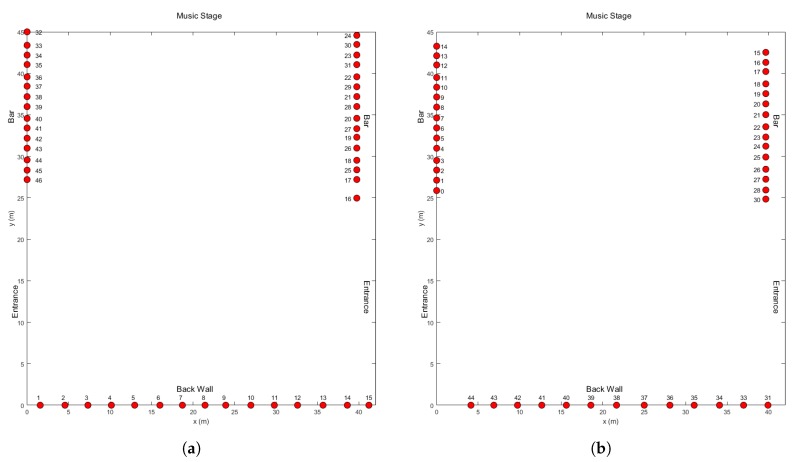
Overviews of the Freedom stage environment for the 2017 and 2018 editions of Tomorrowland, shown respectively in (**a**,**b**).

Schematic overviews of the setups we installed in the approximately 1755 $m^{2}$ Freedom Stage environment in 2017 and 2018 are provided in Figure 24. Type I nodes were installed under the bars of the food and drink stands to the side of the environment and behind the wooden wall in the back. The height at which these nodes were installed was approximately 1.20 m for those placed under the bars and between 0.60 m and 1.00 m for those behind the back wall. This low and variable height was caused by practical considerations related to the manner in which they had to be attached to this wall.

As was the case in previous sections, we entirely disregard the data from links whose lines-of-sight do not intersect the environment. As a result, instead of the 1035 and 903 links implied by the use of 46 and 43 nodes for respectively the 2017 and 2018 experiments, only 705 and 615 links are used.

### Combining Crowd Estimation & Cashless Transaction Data

**Figure 25:**
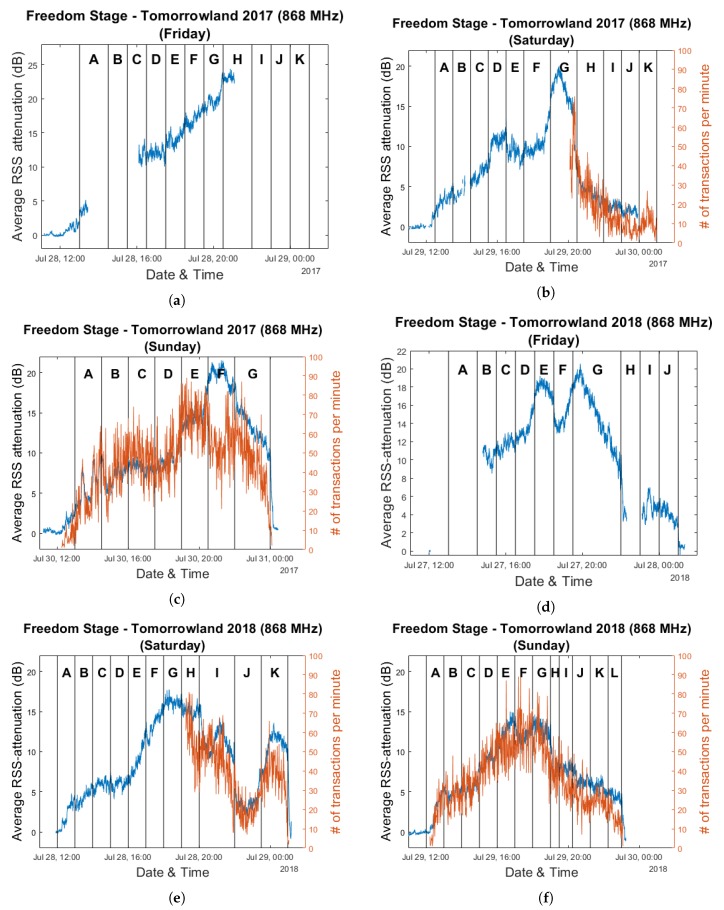
Graphs representing the evolution of the average RSS attenuation and number of cashless transactions per minute for the 2017 and 2018 editions of the Tomorrowland festival within the Freedom Stage environment. Plots (**a**–**c**) are based on data collected on Friday 28 July 2017, Saturday 29 July 2017 and Sunday 30 July 2017. Plots (**d**–**f**) are based on data collected on Friday 27 July 2018, Saturday 28 July 2018 and Sunday 29 July 2018.

The resulting evolution of the average RSS attenuation over the course of the three weekend days is shown in Figure 25 for both years. These graphs are overlaid with the corresponding cashless transaction numbers for the days for which this data was available. As was the case for the Main Comfort analysis graphs, each day is divided into segments based on the performances of the artists which are anonymously labeled by letters A through L.

**Figure 26:**
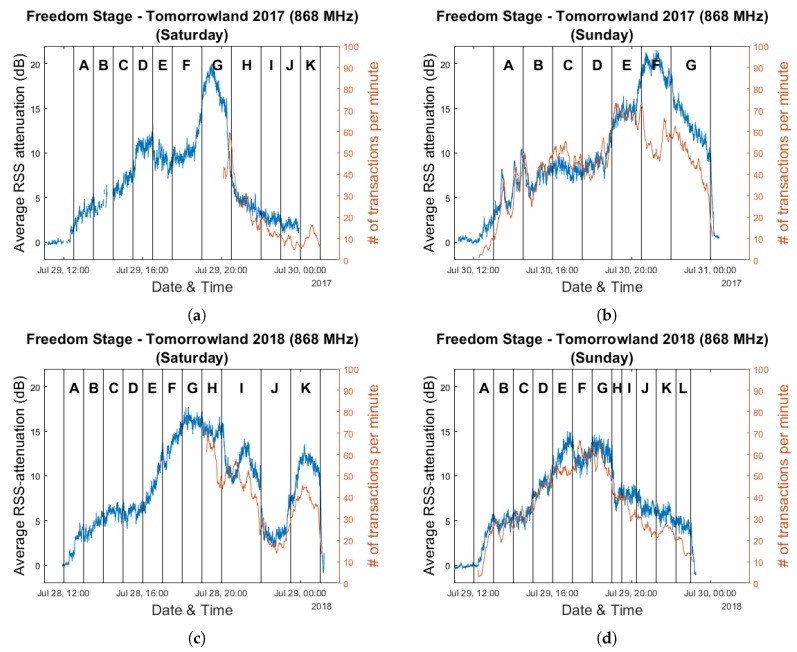
Graphs representing the evolution of the average RSS attenuation and the (smoothed) number of cashless transactions per minute for the 2017 and 2018 editions of the Tomorrowland festival within the Freedom Stage environment. Plots (**a**,**b**) are based on data collected on Saturday 29 July 2017 and Sunday 30 July 2017. Plots (**c**,**d**) are based on data collected on Saturday 28 July 2018 and Sunday 29 July 2018.

**Figure 27:**
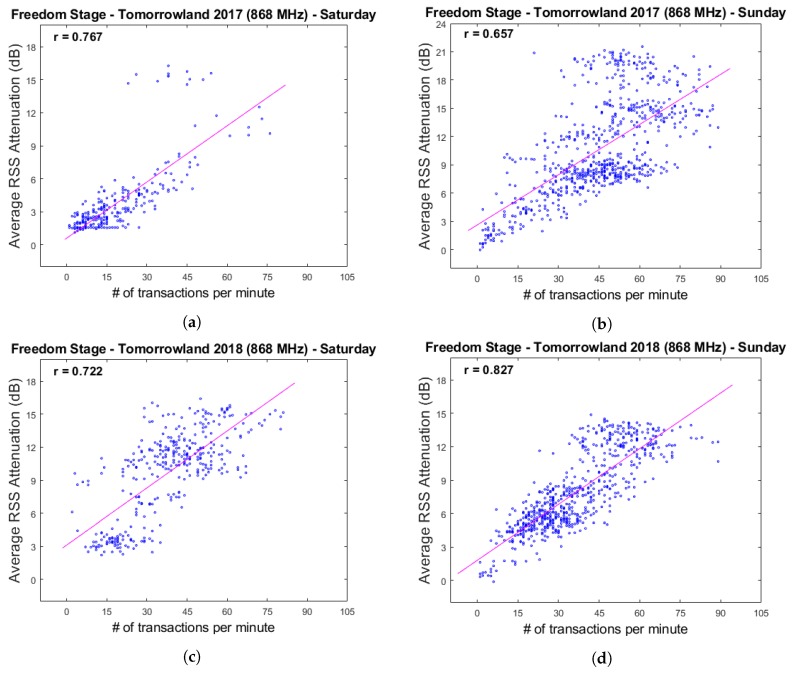
Graphs showing the scatter plots and associated correlation coeffecients for the average rss attenuation and the number of cashless transactions per minute. Plots (**a**,**b**) are based on data collected on Saturday 29 July 2017 and Sunday 30 July 2017. Plots (**c**,**d**) are based on data collected on Saturday 28 July 2018 and Sunday 29 July 2018.

Figure 26 shows the same graphs with transaction data which was smoothed through the use of a moving average filter in order to make the evolution of the data throughout the festival days more clear. Corresponding scatter plots and their associated Pearson correlation coefficients are shown in Figure 27. These were created based on the raw data, as we did not wish to introduce any spurious correlations as a result of a smoothing procedure.

As is clear from these results, decently strong positive correlations exist between these two types of data. One can quite easily observe time intervals in the graphs where—in the short term—the average RSS attenuation and the number of cashless transactions per minute appear to be negatively correlated. For example, this occurs near the end of the performance of artist H on Saturday for Tomorrowland 2018. While the number of transactions strongly decreases, the average RSS attenuation increases. Interestingly, after the end of the performance, the average attenuation sharply lowers as well. If one were to assume that the attenuation metric is strongly correlated with the total amount of people present in the environment—an assumption which we consider to be reasonable given the results described in this paper thus far—a possible explanation could be that event attendees are unlikely to buy food and/or drinks if they are planning to leave after the current artist has finished their performance. Other examples of sudden localized changes in correlation include artist E during Sunday for Tomorrowland 2018 and artist F during Sunday for Tomorrowland 2017. While these are nothing more than a few interesting observations, we believe that this can be an interesting starting point for research regarding the potential for combining an automatic crowd estimation system with other sources of event data to enable real-time crowd prediction. We will discuss this more in detail in the next section.

## 6. Conclusions & Future Work

Our initial study based on measurements performed within the Freedom Stage at the 2017 edition of the music festival Tomorrowland showed that the use of a passive RF sensing approach for large-scale crowd estimation was feasible. In this paper, we validated this result for multiple environments and showed that strong linear correlations existed between the average RSS attenuation experienced by communication links in our RF sensing network and the total amount of people present according to available ground truth data. Furthermore, we made use of straightforward linear regression approaches in which part of the captured RSS data was used for training a crowd estimation model which was then evaluated by applying it to the remaining data. This led to median estimation errors as low as 184 for the Main Comfort environment and 13 for the Nucleus environment. These corresponded to respectively 5.7% and 5.3% of the maximum sizes of the crowds within these environments during the measurement period.

The ground truth data for these environments did suffer from some shortcomings. The bracelet scanning procedure within the Main Comfort for people entering and exiting the environment raised some questions regarding the accuracy of the resulting crowd counts. However, it should be noted that our results were shared with the festival organisers. Well-aware of the potential inaccuracies of the bracelet scanning system, they were impressed by our approach and considered our estimates to be viable. In regards to the Freedom Stage—for which no ground truth data was available—they found the relative evolution of the crowd sizes as indicated by the average RSS attenuation within our RF network to be realistic for the series of artists performing in this environment. While these are naturally non-quantifiable opinions, we nevertheless consider them to be valuable insight from crowd management experts.

As we have indicated multiple times over the course of this paper, we believe that the results obtained from this study have opened up many interesting avenues for further research. First of all, while we hypothesize that the use of the 2.4 GHz frequency band rather than sub-GHz frequencies could pose problems in large-scale environments due to its limited range, experiments need to be performed to determine where its exact limits lie. The use of OFDM-based Wi-Fi signals could enable the use of CSI-measurements, which has already been proven in existing research to be highly useful within small-scale environments [21,22,23]. Additionally, the use of SDR equipment to fully analyze transmitted signals instead of only utilizing RSS as a signal descriptor could be highly interesting as well, although it would evidently not be feasible to replace the large number of nodes we have deployed in the previously described environments with SDR platforms.

This leads directly to a second important future research topic, which is an analysis of the impact of the number of RF nodes. When deploying our nodes in the environments described in this paper, we tended to simply install as many as possible in locations where it was allowed. By virtually removing nodes in an analogous manner to how we removed links whose lines-of-sight did not intersect the environment, we intend to investigate the impact of network size on crowd estimation accuracy in relation to environment size and complexity.

Next, we also wish to delve deeper into the topic of calibration. In our current analysis, we performed new calibration measurements during a short period of time at the beginning of each day. We did so to avoid the impact of small environmental changes (e.g., furniture being moved) that have occurred since the calibration measurements took place. We do not know how large these changes need to be to have any significant impact on crowd estimation accuracy. Additionally, it could cause major issues for a real-time crowd estimation system if severe environmental changes were to occur while an event is ongoing (e.g., extra crowd barriers being installed), due to the fact that recalibration would only be possible after the crowd had left. Furthermore, another interesting research topic related to calibration, is the impact of the battery life cycle on the measured RSS values. Due to the battery run-time for both nodes types being respectively 189 and 147 h and with the described events lasting for at most 3 days, this was not an aspect which came up over the course of the experiments. Nevertheless, this needs to be looked into for future, more long-term setups.

Additionally, more accurate models translating RF network measurements to crowd counts should be developed. Care must be taken to avoid overfitting to one specific environment, however. Exploring the use of other features besides the mean RSS difference (e.g., variance, skew and kurtosis) might prove helpful in this context. Within the current state-of-the-art for small-scale RF based crowd estimation, the use of multiple features is common for both RSS- and CSI-based systems [22,23].

Another topic which opens up a multitude of research directions is the concept of subregions. Dividing the environment into multiple zones can provide information regarding the manner in which the crowd is dispersed throughout the entire environment. The animation detailing the evolution of the average RSS attenuation within each subregion of the Nucleus Tent environment indicates that this concept can be a solid starting point for research regarding crowd flows. Many as of yet open questions will need to be answered over the course of this research. What are the optimal ways to divide the environments into multiple subregions, depending on environment and the number of nodes deployed? Is it feasible to combine more fine-tuned (existing) small-scale crowd estimation techniques for the subregions with more coarse large-scale approaches? How detailed is the crowd flow information which can be derived by using this approach? These are but a few examples.

Crowd activity recognition is also a research field which we would like to explore for large-scale environments. Initially, we are planning on investigating the feasibility of differentiation between a crowd whose members are standing upright versus a crowd whose members are sitting down. It should be noted that for this analysis we will not solely make use of some of the sub-experiments performed during the controlled experiment at Sound of Science 2019. Aside from the controlled experiment we performed at this event, our system was also active during the other talks and presentations given in this environment. While we do not have any verifiable crowd size ground truth corresponding to these measurements, we do know the end times of each talk when people stood up and began to leave the tent. Additionally, we have already performed and are planning to perform even more experimental measurements related to this aspect in a variety of new environments.

Finally, based on certain observations regarding the cashless transaction data and our own measurements in the Freedom Stage environment, we believe that the combination of a passive RF sensing approach with other available data sources at an event could potentially lead to the creation of real-time crowd prediction systems. While the impact of certain factors on the evolution of the crowd size at an event are already well known by event organizers (e.g., artist popularity), quantifying these aspects and incorporating them into prediction models which are continuously updated based on the data from an automatic crowd monitoring system could prove to be an enormously useful tool. In doing so, a crowd estimation approach would form the core of a full-fledged crowd analytics system.

One aspect which would be highly useful to all of the aforementioned research paths, is the creation of publicly available data sets. This is an important issue within the research domain of device-free passive localization in general and we have extensively discussed this topic in [31]. For RF-based passive crowd estimation, the need for data sets is even greater. To the best of our knowledge, no passive RF sensing experiments performed in environments containing thousands or even hundreds of human individuals do exist—with the sole exception of our own. While we acknowledge that it is very difficult to obtain accurate ground truth data for these type of experiments when performed in real-life, uncontrolled environments, the sharing of any and all data—even with imperfect ground truths—would greatly aid the further development of the field. Additionally, one very interesting addition to these data sets would be the inclusion of data from other crowd estimation approaches such as camera images. While the use of cameras does have several important downsides which we touched upon in the introduction, they could still be useful for research into more mid-sized crowds (containing hundreds instead of thousands of human individuals).

To lead by example, we are currently working on an MDPI Data publication in which 3 out of the four data sets discussed in this paper (Main Comfort, Freedom 2017 & Freedom 2018) and the corresponding ground truths and cashless payment amounts will be made public.

## Supplementary Materials

An animation showing the evolution of these maps for the entire experiment is available online at https://www.mdpi.com/1424-8220/20/9/2624/s1.
